# Phase I/II study of erlotinib plus S-1 for patients with previously treated non-small cell lung cancer: Thoracic Oncology Research Group (TORG) 0808/0913

**DOI:** 10.1007/s10637-020-00985-4

**Published:** 2020-08-15

**Authors:** Yoshiro Nakahara, Tsuneo Shimokawa, Yuki Misumi, Naoyuki Nogami, Tetsu Shinkai, Nobuhiko Seki, Yukio Hosomi, Naoya Hida, Hiroaki Okamoto

**Affiliations:** 1 Department of Thoracic Oncology and Respiratory Medicine, Tokyo Metropolitan Cancer and Infectious diseases Center Komagome Hospital, 3-18-22, Honkomagome, Bunkyo-ku, Tokyo, 113-8677 Japan; 2 Department of Respiratory Medicine, Kitasato University School of Medicine, 1-15-1, Kitasato, Minami-ku, Sagamihara, Kanagawa 252-0374 Japan; 3 Department of Thoracic Oncology, Kanagawa Cancer Center, 2-3-2, Nakao, Asahi-ku, Yokohama, Kanagawa 241-8515 Japan; 4 Department of Respiratory Medicine, Yokohama Municipal Citizen’s Hospital, 56 Okazawa-cho, Hodogaya-ku, Yokohama, Kanagawa 240-8555 Japan; 5 Department of Thoracic Oncology, National Hospital Organization Shikoku Cancer Center, 160 Kou Minami-Umemoto, Matsuyama, Ehime 791-0280 Japan; 6 Department of Medical Oncology, Shonan Eastern General Hospital, 500, Nishikubo, Chigasaki, Kanagawa 253-0083 Japan; 7 Division of Medical Oncology, Department of Internal Medicine, Teikyo University School of Medicine, 2-11-1, Kaga, Itabashi-ku, Tokyo, 173-0014 Japan; 8 Department of Respiratory Medicine, Yokohama-City Seibu Hospital of St. Marianna University School of Medicine, 1197-1, Yasashi-cho, Asahi-ku, Yokohama, Kanagawa 241-0811 Japan

**Keywords:** Epidermal growth factor receptor tyrosine kinase inhibitor, Erlotinib, S-1, Non-small cell lung cancer

## Abstract

*Introduction* In preclinical data, the combination therapy with S-1 and epidermal growth factor receptor (EGFR) tyrosine kinase inhibitor (TKI) had a synergistic antitumor effect on non-small cell lung cancer (NSCLC), regardless of the EGFR mutation status. *Patients and Methods* Patients with previously treated NSCLC and adequate organ function regardless of EGFR mutation status were eligible for the phase I study, with wild-type EGFR were eligible for the phase II study. Treatment consisted of erlotinib 150 mg/body orally once every day and S-1 60 mg/m^2^, 70 mg/m^2^, or 80 mg/m^2^ (level 0, level 1, or level 2) orally on days 1–14 every three weeks. The primary endpoint for the phase I study was the determination of the recommended dose (RD), the phase II study was the overall response rate (ORR). *Results* A total of 7 patients with performance-status (PS) 0 or 1 were enrolled as subjects in phase I. Five of these subjects were EGFR-mutation positive. Four subjects were enrolled at S-1 dose level 1 and 3 were enrolled at S-1 dose level 2. No dose-limiting toxicities were observed in these subjects. The RD was decided as erlotinib 150 mg/body and S-1 80 mg/m^2^. In phase I, 5 subjects achieved partial response, and the ORR was 71.4%. A total of 10 patients with PS 0, 1, or 2 EGFR-wild type NSCLC were enrolled in phase II. In phase II, the ORR was 10.0%, and the disease control rate (DCR) was 40.0%. After the enrollment of 10 subjects, enrollment was stopped based on two treatment-related deaths. *Conclusion* The combination therapy of erlotinib plus S-1 was not feasible in the EGFR wild-type NSCLC at least and early stopped. Trial registration: UMIN-CTR Identifier: 000003421 (2010/03/31, phase I), 000003422 (2010/03/31, Phase II).

## Introduction

Lung cancer is the leading cause of cancer deaths worldwide. Effective treatment options for patients with non-small cell lung cancer (NSCLC) whose disease progresses after first-line platinum-based chemotherapy are limited. In the patients without driver oncogene, immune checkpoint inhibitors of anti-programmed death-ligand 1 (PD-L1) and programmed death-1 (PD-1) are now standard second-line treatments [1–4]. For patients who have failed or are not eligible for immunotherapy, docetaxel [5, 6]—with or without ramucirumab [7]—or pemetrexed [8] are other standard therapies in case of relapsed NSCLC.

Erlotinib, an epidermal growth factor receptor (EGFR) tyrosine kinase inhibitor (TKI), showed a significant overall survival benefit in a randomized phase III trial in previously treated advanced NSCLC, regardless of EGFR mutation status [9].

S-1 is an oral fluoropyrimidine-based combination of tegafur, gimeracil, and oteracil potassium in a molar ratio of 1:0.4:1. A randomized phase III study showed non-inferiority of S-1 monotherapy to docetaxel for overall survival in previously treated patients who had undergone at least 1 platinum-based chemotherapy [10]. In that study, significant improvement was shown in the EORTC QLQ-C30 global health status in the S-1 arm.

The combination therapy with S-1 and gefitinib had a synergistic antitumor effect on NSCLC xenografts in vivo, regardless of EGFR mutation status [11]. The synergistic antitumor effect is considered due to Gefitinib-induced down-regulation of thymidylate synthase (TS). Furthermore, Giovannetti E, et al. reported that erlotinib also significantly reduced TS expression and activity, possibly via E2F-1 reduction [12]. Thus, the combination therapy of S-1 and erlotinib might be a promising strategy.

We therefore planned this phase I/II clinical study to evaluate the efficacy and safety of combination therapy of S-1 and erlotinib for previously treated NSCLC.

## Methods

### Patients

#### (Phase I study)

Patients with stage IIIB or IV cytologically or histologically confirmed NSCLC who had disease progression after one or two prior platinum-containing regimens, regardless of EGFR mutation status, were eligible for participation in the study. Eligible patients were 20 years of age or older; had an Eastern Cooperative Group (ECOG) performance-status (PS) score of 0 or 1; at least 1 measurable target lesion; and no prior chemotherapy with EGFR-TKI or fluoropyrimidine.

The criteria for adequate organ function included white blood cell (WBC) count ≥3500 - ≤12,000/μL, neutrophil count ≥2000/μL, platelet count ≥100,000/μL, hemoglobin ≥9.5 g/dL, serum aspirate aminotransferase (AST) and alanine amino transferase (ALT) concentrations ≤2.5 × upper limit of normal (ULN), creatinine level 1.5 × ULN, creatinine clearance ≤60 mL/min, oxygen saturation by pulse oximetry ≥90%.

Patients were excluded from the study if they had interstitial pneumonia or pulmonary fibrosis on chest X-ray, symptomatic brain metastases, massive pleural/pericardial effusion or ascites, or severe concomitant disease.

#### (Phase II study)

Patients with stage IIIB or IV cytologically or histologically confirmed NSCLC with wild-type EGFR who had disease progression after one or two prior platinum-containing regimens were eligible for participation in the phase II study.

An in vitro experiment showed that EGFR wild-type cells are more sensitive to fluorouracil than mutant cells [13]. Based on this data, we decided that the main purpose of the phase II study was to analyze the efficacy of this combination therapy for the patients with wild-type EGFR. Thus, only the patients with wild-type EGFR were enrolled. Eligible patients were 20 years of age or older; had an ECOG PS of 0–2; at least 1 measurable target lesion; and no prior chemotherapy with EGFR-TKI or fluoropyrimidine. Because of the good tolerability observed in the phase I study, PS 2 patients were also enrolled in the phase II study.

The criteria for adequate organ function were almost the same as those of phase I study. However, oxygen saturation by pulse oximetry needed to be ≥94% in the phase 2 section. The exclusion criteria were the same as those of phase I study.

Written informed consent was obtained from every subject. The study protocol was approved by the Thoracic Oncology Research Group (TORG) Protocol Review Committee and the review board of each participating institution.

### Evaluation for enrollment

All patients were required to undergo CT of the thorax and the upper abdomen, either CT or magnetic resonance imaging (MRI) of the brain, and either radioisotope bone scan or positron emission tomography (PET) for the assessment of disease stage. A complete blood cell count and a blood chemistry test were performed at the enrollment.

In the phase II study, EGFR mutation status was analyzed with peptide nucleic acid, locked nucleic acid polymerase chain reaction (PNA-LNA PCR) clamp method, PCR-Invader method (structure-specific 5′ nuclease-based method), direct sequence method, or Scorpion ARMS method.

After the protocol treatment was started, chest X-ray and blood testing were performed at least once a week in the phase1 section, and once per cycle in the phase 2 study. CT was repeated every month to evaluate the target lesions. Tumor response was assessed using the Response Evaluation Criteria in Solid Tumors (RECIST) version 1.1, and toxicity was assessed using the National Cancer Institute Common Terminology Criteria for Adverse Events (CTCAE) version 3.0.

#### Phase I study

**Fig. 1:**
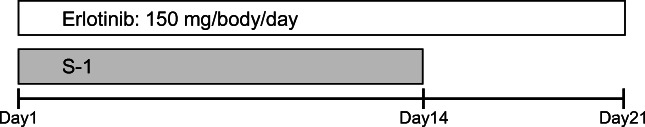
Treatment schedule

The primary endpoint for the phase I study was the determination of the recommended dose (RD). Erlotinib 150 mg/body was administered orally once a day (Fig. 1). S-1 was also given orally, twice daily after meals for 2 weeks in a 3-week cycle. Based on previous studies, the following dose levels of S-1 were evaluated: level 0, 60 mg/m^2^; level 1, 70 mg/m^2^; and level 2, 80 mg/m^2^ (Table 1). S-1 dose was based on body surface area (Table 1). The study treatment was started at level 1.

**Table 1:** Dose of S-1 in phase I and phase II study

Dose escalation schedule of S-1 in phase I study	
Level 2	80 mg/m^2^
Level 1	70 mg/m^2^ (starting dose)
Level 0	60 mg/m^2^
Dose of S-1according to BSA in phase II study	
BSA < 1.25 m^2^	80 mg/day
1.25 m^2^ ≤ BSA < 1.5 m^2^	100 mg/day
1.5 m^2^ ≤ BSA	120 mg/day

*BSA*, body surface area

The dose level was escalated based on the development of toxicity during the treatment, and was not escalated for every subject. Dose-limiting toxicity (DLT) was considered to be any of the following adverse events: grade 4 leukopenia or thrombocytopenia; grade 3 febrile neutropenia; grade 3 non-hematologic toxicity except for nausea, vomiting, anorexia, and alopecia; grade 4 AST/ALT elevation, or grade 3 AST/ALT elevation lasting 7 days or more; serum bilirubin elevation greater than 3.5 mg/dl, or 2.5–3.5 mg/dl lasting 7 days or more; any delay of scheduled oral intake of erlotinib and/or S-1 for 2 weeks or more; or grade 1 ILD with suspected association with the therapeutic drugs. The dose escalation was made, in principle, according to the DLT frequency in the first cycle at a particular dose level. Three subjects were initially enrolled at level 1; if no DLT was observed, then the 3 subjects were to receive level 2 treatment. If 1 of the initial 3 subjects receiving level 1 treatment developed DLT, 3 additional subjects were to be entered at level 1; if 1 of the 6 subjects receiving level 1 treatment developed DLT, then the next subjects were to receive level 2 treatment. If 2 or more subjects of the 6 subjects receiving level 1 treatment developed DLT, we were to define the dose of level 1 as MTD. If all 3 of the initial 3 subjects receiving level 1 treatment developed DLT, we were to define the dose of level 1 as MTD. One level under MTD was to be defined as RD. If the level 1 treatment was MTD, we were to continue this study with level 0 therapy. If no DLT was observed in level 2 treatment, we were to define level 2 dose as RD.

#### Phase II study and statistical analysis

Phase II study was performed based on RD decided in phase I. The primary endpoint of the phase II study was the overall response rate (ORR). Based on the Simon two-stage design, the planned sample size of 50 subjects was determined appropriate to reject a null ORR of 5% at one-sided significance level of 0.05 under an expected ORR of 16% with a power of 0.80. The secondary endpoints were disease control rate (DCR), progression-free survival (PFS), overall survival (OS), and toxicity. The Kaplan–Meier method was used to estimate the median values of time-to-events, such as OS and PFS; and the confidence intervals (CIs) were calculated using the Brookmeyer and Crowley method. All statistical analyses were performed using BellCurve for Excel (Social Survey Research Information, Tokyo, Japan).

## Results

### Baseline characteristics

A total of 7 and 10 subjects were enrolled in phase I and phase II, respectively, from 5 institutions across Japan from October 2010 to April 2012. Enrollment was stopped when 10 subjects were enrolled in phase II study, following the Safety Review Committee’s recommendation based on two treatment-related deaths (TRD). In phase I study, five subjects were EGFR-mutation positive. Subject demographics and disease characteristics are summarized in Table 2.

**Table 2:** Patient characteristics

		Phase I	Phase II	Total
Number		7	10	17
Age (years)	Median	66	60.5	
	(range)	52–70	42–75	
Sex	Male	1	9	10
	Female	6	1	7
Histology	Adenocarcinoma	6	5	11
	Squamous cell carcinoma	0	4	4
	Large cell carcinoma	1	0	1
	Others	0	1	1
ECOG PS	0	2	2	4
	1	5	6	11
	2	–	2	2
EGFR mutation	Positive	5	–	5
	Wild type	1	10	11
	Unknown	1	–	1
Treatment line	Second line	6	3	9
	Third line	1	7	8

*EGFR*, epidermal growth factor receptor

### Phase I MTD and DLT

The phase I study included 7 subjects (Table 3). At level 1, 4 subjects were evaluated, and no subjects developed DLT. The dose was then escalated to level 2, in which 3 subjects were enrolled and treated. At level 2, no subjects developed DLT. Thus, level 2 was considered to be the RD. Toxicities in phase I study are summarized in Table 4.

**Table 3:** Dose escalation in phase I study

Level	Dose		Nubmer of patients	
	Erlotinib (mg/body)	S-1 (mg/m^2^)	Evaluated	With DLT
1	150	70	4	0
2	150	80	3	0

**Table 4:** Toxicities

	Phase I (*n* = 7)					Phase II (*n* = 10)				
	Grade 1/2	Grade 3	Grade 4	All Grade	Grade 3 ≤	Grade 1/2	Grade 3	Grade 4	All Grade	Grade 3 ≤
Decreased appetite	2	0	0	28.6%	0%	7	1	0	80.0%	10.0%
Nausea	3	0	0	42.9%	0%	5	0	0	50.0%	0%
Vomiting	0	0	0	0%	0%	4	0	0	40.0%	0%
Diarrhea	1	0	0	14.3%	0%	7	2	0	90.0%	20.0%
Oral mucositis	4	0	0	57.1%	0%	5	1	0	60.0%	10.0%
Rash/desquamation	6	0	0	85.7%	0%	5	1	0	60.0%	10.0%
Rash/acneiform	3	0	0	42.9%	0%	2	1	0	30.0%	10.0%
Rash/hand-foot skin reaction	1	0	0	14.3%	0%	1	1	0	20.0%	10.0%
Hyperpigmentation	1	0	0	14.3%	0%	2	0	0	20.0%	0%
Dry skin	1	0	0	14.3%	0%	4	0	0	40.0%	0%
Pruritus	2	0	0	28.6%	0%	2	0	0	20.0%	0%
Paronychia	0	0	0	0%	0%	0	1	0	10.0%	10.0%
Fatigue	4	0	0	57.1%	0%	2	0	0	20.0%	0%
Fever	0	0	0	0%	0%	2	0	0	20.0%	0%
Dehydration	0	0	0	0%	0%	0	1	1	20.0%	20.0%
Hypotension	0	0	0	0%	0%	0	0	1	10.0%	10.0%
Hemorrhagic gastric ulcer	0	0	0	0%	0%	1	0	0	10.0%	0%
Colitis	0	0	0	0%	0%	0	0	1	10.0%	10.0%
Sepsis	0	0	0	0%	0%	0	0	1	10.0%	10.0%
Leukocytopenia	2	0	0	28.6%	0%	0	0	0	0%	0%
Neutropenia	2	0	0	28.6%	0%	0	0	0	0%	0%
Anemia	3	0	0	42.9%	0%	5	0	0	50.0%	0%
Thrombocytopenia	1	0	0	14.3%	0%	1	1	0	20.0%	10.0%
Hyperbilirubinemia	4	1	0	71.4%	14.3%	5	0	0	50.0%	0%
Aspartate aminotransferase elevation	2	0	0	28.6%	0%	1	0	0	10.0%	0%
Alanine aminotransferase elevation	1	1	0	28.6%	14.3%	1	0	0	10.0%	0%
Alkaline phosphatase elevation	1	0	0	14.3%	0%	2	0	0	20.0%	0%
Creatinine elevation	2	0	0	28.6%	0%	2	0	0	20.0%	0%

### Efficacy

In phase I study, among 7 subjects, 5 subjects achieved partial response (PR), 1 subject had stable disease (SD), and 1 subject had progressive disease (PD). Thus, the ORR was 71.4%, and the disease control rate (DCR) was 85.7%. In phase II study, among 10 subjects, 1 subject achieved partial response (PR), 3 subjects had stable disease (SD), 3 subjects had progressive disease (PD), and 3 subjects were not evaluable (NE). Thus, the ORR was 10.0%, and the disease control rate (DCR) was 40.0%. The data regarding efficacy are summarized in Table 5.

**Table 5:** Treatment efficacy

	Phase I	Phase II	Total
	(*n* = 7)	(*n* = 10)	(*n* = 17)
CR	0	0	0
PR	5	1	6
SD	1	3	4
PD	1	3	4
NE	0	3	3
RR	71.4%	10.0%	35.3%
DCR	85.7%	40.0%	58.8%

*CR* complete response, *DCR* disease control rate, *NE* not evaluable, *PD* progressive disease, *PR* partial response, *RR* response rate, *SD* stable disease

### Toxicities in phase II study

Toxicities in phase II study are summarized in Table 4. The protocol treatment was stopped in 4 subjects due to adverse effects: grade 3 diarrhea in 2 subjects, hyperbilirubinemia (total bilirubin: 3.5) in 1 subject, and grade 3 dehydration in 1 subject. There were two TRDs: 1 subject died because of acute respiratory failure on day 65, caused by pulmonary embolism based on pathological anatomy; 1 subject died because of sepsis due to diarrhea and bacterial enteritis on day 25. Enrollment was stopped, following the recommendation of the Safety Review Committee based on these two TRDs.

### Treatment cycle

Treatment cycles in phase I and phase II studies are shown in Table 6. In phase I study, 3 subjects (43%) continued more than 6 cycles (6, 12, and 17 cycles). On the other hand, in phase II section, 5 subjects (50%) discontinued protocol treatment after the 1st cycle. Only 1 subject (10%) continued more than 6 cycles (8 cycles).

**Table 6:** Treatment cycles

	Phase I	Phase II	Total
	(*n* = 7)	(*n* = 10)	(*n* = 17)
1 cycle	2	5	7
2 cycles	1	0	1
3 cycles	1	3	4
4 cycles	0	1	1
5 cycles	0	0	0
6 cycles	1	0	1
7 cycles	0	0	0
More than 8 cycles	2	1	3

## Discussion

This phase II study in patients with previously treated NSCLC with wild type EGFR revealed that the combination therapy of erlotinib and S-1 was not feasible, and the enrollment was stopped, following the recommendation of the Safety Review Committee based on two TRDs.

The cause of difference between toxicities in the phase I study and the phase II study might have been due to the difference in study populations in phase I and phase II. In phase I, the inclusion criteria allowed enrollment of EGFR-mutated patients, and 5 EGFR-mutated subjects and no subjects with squamous cell carcinoma were enrolled. Furthermore, the inclusion criteria allowed only PS 0 and 1 patients, and 6 subjects were enrolled as second-line setting. These subject characteristics led to the high efficacy and tolerability observed in the phase I study. Kiyota, et al. reported good tolerability of gefitinib and S-1 combination therapy [14]. In that study, subjects with only adenocarcinoma were enrolled, and all subjects enrolled in that study were PS 0, or 1. On the other hand, in the phase II of our study, the inclusion criteria did not allow enrollment of EGFR-mutated patients; all subjects were EGFR wild type, and 4 subjects with squamous cell carcinoma were enrolled. Several trials showed that the efficacy of erlotinib for non-adenocarcinoma [9, 15], or smokers [9, 16] was poor. Furthermore, in the phase II of our study, the inclusion criteria allowed PS 0–2 patients; 2 PS 2 subjects were enrolled, and 7 subjects were enrolled as third-line setting. These subject characteristics led to the low efficacy and strong toxicity observed in the phase II of our study. In our study, although RD was decided appropriately in phase I study, due to the differences in inclusion criteria, serious adverse events frequently occurred in phase II study.

Treatment for cancer now can be individualized based on the molecular testing profile of the cancer. Many trials showed that EGFR-TKIs have remarkable efficacy in patients with EGFR activating mutations. These genotyping-guided treatments have been effective in clinical practice. Along with these trials, the role of EGFR-TKIs in patients with wild-type EGFR had been discussed. The Tarceva Italian Lung Optimization Trial (TAILOR) was a randomize phase III trial that compared erlotinib and docetaxel as second-line treatment of patients with advanced NSCLC with wild-type EGFR [17]. That trial showed that docetaxel superior to erlotinib in terms of overall survival (OS), progression-free survival (PFS), and response rate (RR). Furthermore, a subgroup analysis of a Japanese randomized phase III trial showed that PFS and RR with docetaxel were significantly better than those of erlotinib in patients with wild-type EGFR [15]. In that analysis, though not statistically significant, OS of docetaxel tended to be better than that of erlotinib. From these data, in patients with wild-type EGFR, the efficacy of erlotinib is seen to be limited.

Several trials of erlotinib and cytotoxic chemotherapy combination have been performed. However, in those trials, the combination therapy did not confer a survival advantage over cytotoxic chemotherapy alone in unselected patients [18, 19]. On the other hand, in EGFR-mutant NSCLC, the combination therapy of EGFR-TKI and cytotoxic chemotherapy showed promising efficacy. Some clinical trials that analyzed the combination of EGFR-TKI and pemetrexed were reported. The JMIT study, an open-label randomized phase II study, compared the combination therapy of gefitinib and pemetrexed versus gefitinib monotherapy [20], and showed the median PFS with gefitinib and pemetrexed was significantly longer than that with gefitinib alone [15.8 vs 10.9 months; adjusted Hazard Ratio (HR) 0.68; 95% CI, 0.48–0.96; *P* = .029]. Study NEJ009, a randomized phase III study, compared gefitinib monotherapy with gefitinib plus pemetrexed and carboplatin, and showed that gefitinib plus pemetrexed and carboplatin demonstrated better ORR and PFS than the gefitinib monotherapy (ORR, 84% v 67% [*P* < .001]; PFS, 20.9 v 11.9 months; HR for death or disease progression, 0.490 [*P* < .001]) [21]. In that study, median OS in the gefitinib plus pemetrexed and carboplatin group was also significantly longer than in the gefitinib monotherapy group (50.9 v 38.8 months; HR for death, 0.722; *P* = .021). Furthermore, another randomized phase III study of the same design also showed that gefitinib plus pemetrexed and carboplatin demonstrated better ORR, PFS, and OS than the gefitinib monotherapy (ORR, 75% v 63% [*P* = .01]; PFS, 16 v 8 months; HR for death or disease progression, 0.51 [*P* < .001]; OS, not reached v 17 months; HR for death, 0.45 [P < .001]) [22]. Based on these data, in patients with EGFR-mutant NSCLC, the combination therapy of pemetrexed and EGFR-TKI would seem promising. The rationale for combining pemetrexed and EGFR-TKI is the suppression of TS by EGFR-TKI. Therefore, it was concluded that combination therapy of erlotinib and S-1 might be effective for EGFR-mutant NSCLC.

The main limitation of the present study was that it was a clinical trial with small sample size. Also, since many trials showed that the efficacy of erlotinib was limited in patients with wild-type EGFR, the significance of combination therapy in unselected patients had faded. Furthermore, other, more effective drugs such as immune checkpoint inhibitors have been established as the standard treatments based on large phase III studies, and thus the expectations for the combination therapy of erlotinib plus S-1 had declined.

In conclusion, the combination therapy of erlotinib and S-1 was not feasible in the EGFR wild-type NSCLC at least and early stopped.

## Data Availability

The data and materials used in this study are available from the corresponding author on reasonable request.
